# Assertive Community Treatment for alcohol dependence (ACTAD): study protocol for a randomised controlled trial

**DOI:** 10.1186/1745-6215-13-19

**Published:** 2012-02-20

**Authors:** Helen Gilburt, Tom Burns, Alex Copello, Simon Coulton, Michael Crawford, Ed Day, Paolo Deluca, Christine Godfrey, Steve Parrott, Abigail K Rose, Julia MA Sinclair, Christine Wright, Colin Drummond

**Affiliations:** 1 Department of Addictions, Institute of Psychiatry, Kings College London, London, UK; 2 Department of Psychiatry, University of Oxford, Oxford, UK; 3 School of Psychology, University of Birmingham, Birmingham, UK; 4 Centre for Health Services Studies, University of Kent, Canterbury, UK; 5 Faculty of Medicine, Imperial College London, London, UK; 6 School of Psychiatry, University of Birmingham, Birmingham, UK; 7 Department of Health Sciences, University of York, York, UK; 8 Institute of Psychology, Health and Society, University of Liverpool, Liverpool, UK; 9 Faculty of Medicine, University of Southampton, Southampton, UK; 10 Department of Mental Health, St George's, University of London, London, UK

**Keywords:** assertive outreach, alcohol dependence, case management, substance use treatment, assertive community treatment

## Abstract

**Background:**

Alcohol dependence is a significant and costly problem in the UK yet only 6% of people a year receive treatment. Current service provision based on the treatment of acute episodes of illness and emphasising personal choice and motivation results in a small proportion of these patients engaging with alcohol treatment. There is a need for interventions targeted at the population of alcohol dependent patients who are hard to engage in conventional treatment. Assertive Community Treatment (ACT), a model of care based on assertive outreach, has been used for treating patients with severe mental illnesses and presents a promising avenue for engaging patients with primary alcohol dependence. So far there has been little research on this.

**Methods/Design:**

In this single blind exploratory randomised controlled trial, a total of 90 alcohol dependent participants will be recruited from community addiction services. After completing a baseline assessment, they will be assigned to one of two conditions: (1) ACT plus care as usual, or (2) care as usual. Those allocated to the ACT plus care as usual will receive the same treatment that is routinely provided by services, plus a trained key worker who will provide ACT. ACT comprises intensive and assertive contact at least once a week, over 50% of contacts in the participant's home or local community, and comprehensive case management across social and health care, for a period of one year. All participants will be followed up at 6 months and 12 months to assess outcome post randomisation. The primary outcome measures will be alcohol consumption: mean drinks per drinking day and percentage of days abstinent measured by the Time Line Follow Back interview. Secondary outcome measures will include severity of alcohol dependence, alcohol related problems, motivation to change, social network involvement, quality of life, therapeutic relationship and service use. Other outcome variables are treatment engagement including completion of assessment, detoxification and aftercare.

**Discussion:**

Results of this trial will help clarify the potential beneficial effects of ACT for people with alcohol dependence and provide information to design a definitive trial.

**Trial registration number:**

ISRCTN: ISRCTN22775534

## Background

The use of alcohol is one of the leading contributors to ill-health and premature deaths, and is the third leading cause of disability in Europe [1]. In England approximately 4% of the population aged 16-65 are alcohol dependent [2], but access to services is limited with large regional variations. The cost of alcohol misuse to the UK economy is estimated to be £25 bn [3] and over £200 m is spent annually on alcohol treatment in England. People with alcohol dependence who are not engaged in treatment use disproportionate levels of health, social, and criminal justice services, often through unplanned care [4].

The Alcohol Harm Strategy for England highlighted the need to improve treatment services for people with alcohol dependence, particularly those with more complex needs [5] and Models of Care for Alcohol Misusers (MoCAM) supported the development of specialist delivered interventions for this population [6]. Current alcohol service provision focuses on discrete, time-limited episodes of intervention, typically of a few weeks to a few months duration and emphasises personal choice and motivation. However studies have shown that engagement is often problematic [7,8], and despite conventional treatment approximately one third of alcohol dependent patients continue to drink heavily and have poor long term outcomes [9]. For this group of patients, alcohol dependence is a chronic, relapsing disorder [10-12] and in typical practice, treatment often involves multiple episodes extending over many years.

A parallel has been drawn between the management of chronic relapsing alcohol dependence and the management of various chronic physical and mental health problems [13]. In these a 'chronic disease management' approach has become an accepted clinical model in some chronic conditions such as diabetes. This involves ongoing care extending over a prolonged period to alter the long term trajectory of the disease. In adult mental health care, ACT is an extensively researched and widely used model of care for treating unmotivated and difficult to engage patients with severe and enduring mental illness. It emphasises active engagement over an extended period [14,15]. Key features of effective ACT include: (i) rapid access to services, (ii) a small caseload, (iii) a high ratio of community to office-based appointments, (iv) assertive engagement (e.g. with multiple attempts) and (v) a shared care approach, with care coordinators working within a multidisciplinary team that meets frequently [16,17].

In the United States ACT has been shown to be effective in reducing the number and length of admissions to hospital and achieving stable accommodation. It improves patients' satisfaction and motivation for treatment [18,19], and is effective in retaining patients in care [20,21]. European studies, however, have been less supportive of ACT and no advantage over usual care has been found for clinical or social outcomes, although it has been demonstrated to improve client engagement and satisfaction with services [22]. One reason identified for the lack of ACT superiority observed in the UK is that usual care for severe mental illness duplicates several of the key elements of ACT [16,22]. No such overlap is apparent in the current treatment of alcohol dependence. Some of the features of ACT have already been individually applied to the treatment of alcohol dependence and found to improve aspects of outcome. For example, [23] used home visits as part of an aftercare package and found that these increased the likelihood of treatment completion, while a randomised controlled trial found that a flexible and extended approach to aftercare increased the time to first drink and reduced the severity of relapse [24,25]. Most recently a pilot of assertive engagement methods resulted in a significantly greater number of patients completing treatment and entering aftercare [7]. However, the potential benefits of elements of ACT in patients with alcohol dependence but without severe mental illness remain unclear as are the specific aspects of the model that might apply to this disorder.

In this article we describe the methods of an exploratory randomised controlled trial (RCT) that is designed to test the feasibility of implementing ACT for people with chronic relapsing alcohol dependence. It aims to identify both optimal and problematic elements of ACT specific to this population and to establish the potential effect size of ACT versus standard care and evaluate 'proof of principle'.

## Methods/Design

### Overview

The Assertive Community Treatment for Alcohol Dependence (ACTAD) study is a two centre single blind randomised controlled trial of assertive outreach plus care as usual (CAU) compared with a CAU control condition for people with alcohol dependence. The primary analysis will be intention-to-treat.

### Intervention

#### Assertive community treatment intervention

ACT was originally developed for patients with psychotic disorders and has become an established model of care for this population in the US and UK. Since this intervention has never been used for people with primary alcohol dependence, the research team will implement an ACT model taking into account the original model and recent research identifying effective elements in UK studies. A treatment manual will be developed by the research team together with experts in the provision of ACT and incorporating existing research evidence.

The ACT intervention will comprise the following:

(i) Maximum caseload of 15 ACT patients per ACT practitioner

(ii) Input from a multidisciplinary team (including psychiatrists, substance misuse specialists)

(iii) Regular contact (minimum of once a week; 50% of contacts outside of the service settings, either in the patients' home or neighbourhood; short frequent contacts rather than long complex contacts will be encouraged).

(iv) Assertive engagement - persistent and repeated attempts to contact, emphasis on maintaining contact and building relationships

(iii) Focus on both health and social care needs - including accommodation, leisure, occupation, and physical and mental health

(iv) Flexibility - practitioners should work flexibly with patients' goals even when these are peripheral to the addiction.

(v) Openness - practitioners are explicit about their goals both in care planning and in visits.

(viii) Going out of your way - stepping outside of professional roles and going the extra mile for patients.

(ix) Extended care - provided for a prolonged period of 1 year.

#### Training

The training will comprise a three day workshop and a one day clinical placement. The workshop will provide specialist addiction clinicians with information about ACT, its' history, implementation in mental health and the ACTAD trial. Following the workshops each practitioner will attend a one day placement with a mental health assertive outreach team. They will shadow a member of staff including attending community and home visits with patients and have the opportunity to ask questions regarding the day to day practice of providing ACT. A final training day will focus on ACT practice in addictions. A minimum of three members of staff at each site will receive the training.

### Control intervention

The participants in the control condition will receive CAU, as this provides an appropriate comparison with routine clinical practice and will address the issue of whether ACT plus CAU is more effective than CAU alone. CAU components will be actively recorded as part of the trial but are likely to include allocation of a keyworker when one becomes available, with subsequent contact as required. All contact is usually conducted at the service via an appointment based system. Participants will receive a full assessment of alcohol, social and physical health needs, and a risk assessment. CAU focuses primarily on alcohol use and promoting abstinence and relapse prevention. It includes access to medical detoxification, psychological interventions focused on drinking behaviour and available aftercare as required. CAU may also include input from specialists in addictions psychiatry, clinical psychology and social work where available. Patients are most often signposted to other relevant agencies when these services are not directly provided by the addiction treatment service. Aftercare will typically be provided outside the community addiction services. The majority of patients are discharged to primary care within 12 weeks of being allocated a keyworker unless there are significant risks identified. Failure to attend appointments also results in discharge from the service. To establish the variability of CAU for a full-scale trial, interviews will be undertaken with team leaders at each study site and an additional four national sites examining care pathways.

### Intervention fidelity

Each ACT practitioner will participate in a training course involving a series of workshops and placements prior to delivering the intervention to eligible patients. During the intervention, practitioners are encouraged to attend monthly supervisory meetings with the research team during which fidelity will be assessed and the ACT intervention will be reinforced. In addition, staff providing care to participants in both arms of the trial will complete a contact log detailing the care they provide for each patient following each contact. The log, developed from a previous study of assertive outreach in the UK [17], includes details about the mode of contact (i.e. face to face, telephone), setting, focus of contact, and the member of staff involved. This will provide a measure of the care provided to participants in each arm of the trial.

### Participants and baseline recruitment

Potential participants will be identified by staff in participating community addiction services. In services operating a triage or self-referral system, patients presenting with alcohol problems will be screened by staff on presentation. In services operating a referral only system, all referrals will be screened by a member of the clinical team. The team will identify patients in the first instance with a primary alcohol disorder, previous contact with participating addiction services for alcohol dependence, and no history of violence to treatment staff or risk to others as determined by registration under the UK Multi-Agency Public Protection Arrangement (MAPPA). All patients meeting these criteria will be contacted and asked if they would like more information about the research trial and asked for verbal consent to being contacted by a member of the research team.

Research workers will approach patients who have expressed an interest in the study and invite them to a meeting in order to discuss the study in detail. At this meeting the researchers will undertake a full screening of eligibility. Eligible patients will be given a full description of the study and provided with a written information sheet. If willing to participate, they will be invited to sign the consent form. A baseline interview will then be conducted by the research worker taking between 1-11/2hrs. Subsequently, the patient will be randomised using a secure, remote randomisation service independent of the research team, to either the intervention or control group.

**Figure 1:**
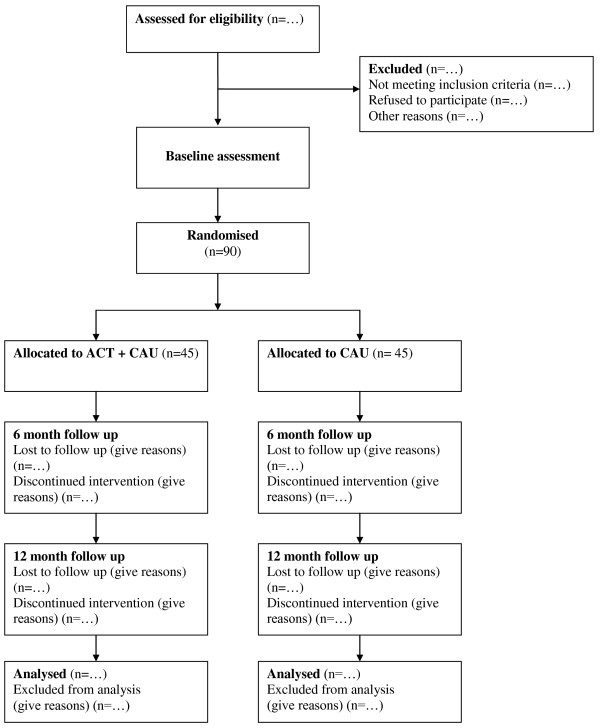
**Participant flow diagram**. The participant flow diagram illustrates randomisation of 90 patients with alcohol dependence to ACT plus CAU or CAU, consistent with the Consolidated Standards of Reporting Trials (CONSORT) 2010 statement.

Figure 1 shows a participant flow diagram for the trial, consistent with the Consolidated Standards of Reporting Trials (CONSORT) 2010 statement [26]

### Eligibility

#### Inclusion criteria

i) Age18 years or over

ii) Is able to understand English sufficiently well to obtain informed consent and complete the assessment instruments

iii) Has attended an NHS Community addiction service in either of the participating Trusts for alcohol dependence on at least one previous occasion in the last five years

iv) Is alcohol dependent. The Composite International Diagnostic Interview [27] is used to establish dependence, a diagnosis characterised by craving, tolerance, a preoccupation with alcohol and continued drinking in spite of resulting harmful consequences [28].

#### Exclusion criteria

i) Unable to give informed consent

ii) Is street homeless

iii) Is diagnosed with a psychotic disorder

iv) Is in receipt of assertive outreach services or has community mental health team (CMHT) input once or more a month

v) Has a severe cognitive impairment as determined by Mini Mental State Examination score of ≤ 10 [29]

vi) Has a history of violence to staff or is registered under MAPPA

### Assessments

#### Baseline assessments

(i) Socio-demographic details - age, sex, ethnicity, marital status, living arrangement, children, living arrangements of children, education

(ii) Drinking in the previous 90 days - Timeline Followback form 90I [30]

(iii) History of drug use - Timeline Followback

(iv) Alcohol related problems - Alcohol Problems Questionnaire [31]

(v) Severity of alcohol dependence -Severity of Alcohol Dependence Questionnaire [32]

(vi) Health utility - EQ-5D [33]

(vii) Health Related Quality of life - SF-12 [34]

(viii) Motivation to change - Readiness to Change Treatment version [35]

(ix) Social network involvement - Important People and Activities Inventory [36]

(x) Health service utilisation - York Service Use Questionnaire

#### Follow up assessments

Six months and 12 months after randomisation, all participants will be followed up. A research worker, blind to treatment allocation, will arrange a convenient time to meet with the participant in order to complete the following measures:

(i) Changes in socio-demographic details - change in relationship status, living arrangements, and living arrangements of children

(ii) Timeline Followback form 90I

(iii) Drug use in previous 90 days - Timeline Followback

(iv) Alcohol Problems Questionnaire

(v) Severity of Alcohol Dependence Questionnaire

(vi) EQ-5D

(vii) SF-12

(viii) Readiness to Change

(ix) Important People and Activities Inventory

(x) York Service Use Questionnaire

(xi) Therapeutic relationship - STAR [37]

### Randomisation and blinding

Randomisation is performed after the baseline assessment. Randomisation is stratified by severity of alcohol dependence (< = 30 and > 30), as measured by the Severity of Alcohol Dependence Questionnaire (SADQ), and by site. The University of York Trials Unit will perform remote randomisation. Confirmation of eligibility, consent, and baseline data will be obtained prior to randomisation. Research workers conducting follow up assessments are blind to the allocated intervention. All possible attempts at maintaining blinding will be pursued.

### Sample size and power calculation

As this study is the first to examine the effect size of ACT in alcohol dependence, there is no specific study on which to base the power calculation. In terms of alcohol consumption a proportional difference between the groups of 30% would be considered a clinically important difference. This equates to an effect size difference of 0.70 similar to the effect sizes found in Patterson et al study [38] and the Cochrane meta-analysis of ACT [19]. As a pilot study we have employed an alpha of 0.2 and power of 80%, a sample of 35 in each group followed-up at 12 months would allow us to detect an effect size of the order of 0.5, a more conservative estimate of effect than seen in similar studies. Allowing a loss to follow-up of 25% would suggest we require 45 in each group, a total of 90 participants. This number would allow us to estimate the potential effect, within 80% confidence intervals in order to enable more formal sample size calculations for a definitive study.

### Statistical analysis

The main hypothesis, stated as a null hypothesis, is that CAU augmented with ACT is no more effective than CAU in reducing alcohol consumption 12 months after randomisation. The primary outcome measures will be alcohol consumption: mean drinks per drinking day and percentage of days abstinent at 12 months measured using the Timeline Followback interview. The primary analysis will be intention-to-treat in which participants will be analysed as part of their allocated group irrespective of the treatment received; this provides the most rigorous estimate of effectiveness. The primary outcome measure will be analysed using an analysis of covariance approach adjusting for known confounding variables such as baseline mean drinks per drinking day, age and gender. If the assumptions underlying ANCOVA are not met transformations will be undertaken and if transformations are not viable alternative non-parametric approaches will be used. In order to explore the impact of missing data on the primary outcome measure we will employ multiple imputation techniques and conduct sensitivity analyses to measure any effect of missing data on reported outcomes.

Continuous secondary measures will be analysed in a similar manner. Categorical variables will be analysed using chi-squared statistics and binary outcomes analysed using logistic regression controlling for known confounding variables. Estimates and the 80% confidence intervals will be presented. In addition intra-class correlation coefficients relating to therapists will be calculated and estimates of recruitment, engagement in treatment, retention will be assessed to inform any future study.

### Economic evaluation

Resources used in care provision will be recorded on an individual patient basis by staff providing ACT and CAU for each patient following every contact and enable further costing of care provision. The main economic evaluation will take an NHS perspective, including the cost of all hospital and community health and social care services, as recommended by the National Institute for Health and Clinical Excellence (NICE). A wider perspective will also be adopted by measuring and applying unit costs to contacts with the criminal justice system. Resource use information will be collected using a modified version of the York Adult Service Use Schedule. Unit costs of wider health care utilisation beyond the trial interventions will be compiled from a variety of sources including NHS Reference Costs [39] and the Unit costs of Health and Social Care published by the Personal Social Services Research Unit (PSSRU) [40]. EQ-5D will be used to estimate Quality Adjusted Life Years (QALYs) and the changes in QALYs for ACT and CAU will be combined with cost data to perform an incremental cost-effectiveness analysis. Patient level cost and outcome data will be bootstrapped and cost-effectiveness acceptability curves will estimate the probability that ACT is more cost-effective than CAU over a range of threshold values at which the decision maker is willing to pay for a QALY.

### Process evaluation

Since this is the first time ACT has been provided for patients with a primary diagnosis alcohol dependence, a process evaluation will be undertaken. This will explore the processes through which ACT works in practice, staff and participant experiences of ACT, and aid in the identification of optimal and problematic elements of ACT in this population.

ACT practitioners will complete a case report for each participant after the 12 month intervention period. The case reports will detail presenting problems, care provided over the 12 month period and the main outcomes.

Participants will be purposively sampled to include positive and negative drinking outcomes such as abstinence and no change in drinking, engagement and non-engagement with the ACT practitioner and intervention, and positive and negative experiences of providing and receiving ACT. Each ACT recipient identified will be invited to take part in a semi-structured interview following their 12 month follow up quantitative interview. This will explore how the participant came into the trial, their experience of ACT during the treatment period including identifying helpful and unhelpful elements, their perceptions of change during this period, and problems that have not been addressed.

Participants will also be asked to compare this episode of care with previous episodes, and identify any other factors (external to the ACT) that may have had an impact on their drinking during the intervention period. Approximately 12 participants will be interviewed across all three sites. A parallel interview will be undertaken with the participants' ACT practitioner. The practitioner interview will explore the process and content of ACT care provision from initial presentation following randomisation through the 12 month intervention period. As with the ACT recipient interview, practitioners will be asked to identify elements of care that have been helpful or problematic, perceptions of change and areas that they have been unable to address to date. The participant and ACT practitioners interviews will form paired data sets enabling triangulation of data.

Finally, an interview will be undertaken with each of the ACT practitioners to explore their overall experience of providing ACT, taking into account all participants they have provided care for as part of the trial. These interviews will be analysed in conjunction with the paired interviews providing further detail on the process and content of ACT care provision, as well as differences between individual practitioners and research sites.

To enhance the understanding of CAU, a further 12 interviews across the sites will be undertaken with participants who were randomised to CAU alone. Participants will purposively sampled using the same criteria as for participants interviewed in the ACT arm of the trial. Similarly, the interview will explore how the participant came into the trial, their experience of CAU during the treatment period including identifying helpful and unhelpful elements, their perception of change during this period, and problems that have not been addressed. Additionally participants will be asked to identify any other factors external to CAU that may have had an impact on their drinking during the study period.

### Ethics

The study protocol was approved by National Research Ethics Service Committee London - Chelsea (REC number: 08/H0801/113).

## Discussion

ACT has gained wide acceptance in the US and the UK as a model of care for people with severe and enduring mental illness. It focuses on the delivery of effective interventions in addition to addressing the wider social and health needs of patients through assertive, intensive and flexible engagement. ACT has been demonstrated to reduce the frequency and length of psychiatric admissions while increasing social functioning, and proven to be cost effective for those with long term relapsing conditions.

UK studies have shown less benefits of ACT over standard care and it has been suggested that this may be in part due to the enhanced nature of standard care in mental health provision. In contrast to care for mental health problems, current provision for alcohol dependence is time-limited and focused on treatment of alcohol dependence rather than accompanying problems. Assertive Community Treatment therefore offers a potential advantages for engaging patients in treatment, delivering effective interventions and maintaining abstinence over a period of time for those whom current provision has failed. The ACTAD study is the first clinical trial to examine the role of ACT in treatment of primary alcohol dependence. Since previous studies of ACT in mental health have suggested that both treatment fidelity and the level of standard care may be importance, ACT fidelity will be measured and CAU will be described. Finally, a comprehensive process evaluation will seek to establish application of ACT for alcohol dependence and issues surrounding implementation to inform a definitive trial.

## Trial status

The trial is currently in the recruitment phase.

## Competing interests

The authors declare that they have no competing interests.

## Authors' contributions

CD, AR and SC developed the original concept of the trial; CD, AR, SC and PD drafted the original protocol and developed the design and methodology; CG and SP developed the health economic component; HG and JS developed the process measurement component; CD, TB, HG, CW, MC, ED and AC developed the ACT intervention; SC advised on the trial design and methods; HG and CD adapted the trial proposal as a protocol paper; all authors reviewed and commented on the final manuscript.
